# Crystal structure of ({(1*R*,2*R*)-*N*,*N*′-bis­[(quino­lin-2-yl)methyl]cyclo­hexane-1,2-di­amine}­chlorido­iron(III))-μ-oxido-[tri­chlorido­ferrate(III)] chloro­form monosolvate

**DOI:** 10.1107/S2056989017007952

**Published:** 2017-06-02

**Authors:** Hannah Swift, Molly W. Carrig, Kayode D. Oshin, Anastasiya I. Vinokur, John A. Desper, Christopher J. Levy

**Affiliations:** a Department of Chemistry, Creighton University, Omaha, NE 68102, USA; b Department of Chemistry, University of Wisconsin, Madison, WI 53558, USA; c Department of Chemistry, Kansas State University, Manhattan, KS 66506, USA

**Keywords:** crystal structure, six-coordinate iron(III) complex, bridging oxido ligand, tetra­dentate ligand, chiral ligand

## Abstract

The first Fe^III^ atom in the solvated title compound, [Fe_2_Cl_4_O(C_26_H_28_N_4_)]·CHCl_3_, adopts a distorted six-coordinate octa­hedral geometry. It is coordinated by one chloride ligand, four N atoms from the (1*R*,2*R*)-*N*,*N*′-bis­[(quinolin-2-yl)methyl]cyclo­hexane-1,2-di­amine ligand, and a bridging oxido ligand attached to the second Fe^III^ atom, which is also bonded to three chloride ions. A very weak intra­molecular N—H⋯Cl hydrogen bond occurs. In the crystal, the coordination complexes stack in columns, and a grouping of six such columns create channels, which are populated by disordered chloro­form solvent mol­ecules. Although the Fe—Cl bond lengths for the two metal atoms are comparable to the mean Fe—Cl bond lengths as derived from the Cambridge Structural Database, the Fe—O bond lengths are notably shorter. The solvent chloro­form mol­ecule exhibits ‘flip’ disorder of the C—H moiety in a 0.544 (3):0.456 (3) ratio. The only directional inter­action noted is a weak C—H⋯Cl hydrogen bond.

## Abstract 2

The bimetallic title compound contains an Fe^III^ center coordinated by one chloride ligand, four N atoms and a bridging oxo ligand in a distorted octa­hedral geometry. The bridging oxo ligand is connected to a second Fe^III^ atom with three coordinating chloride ligands.

## Chemical context   

**Figure 1:**
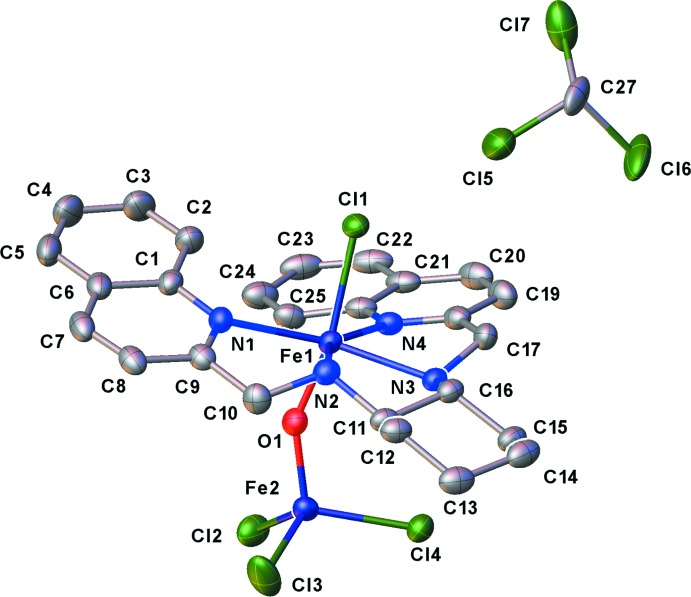
The mol­ecular structure of complex (**1**), shown with 50% probability displacement ellipsoids. All H atoms and the minor-disorder components of the solvent mol­ecule have been omitted for clarity.

Developing small-mol­ecule complexes incorporating iron is an area of growing inter­est since the discovery of non-heme iron enzymes such as methane monooxygenase and Rieske di­oxy­genases to be efficient catalysts in the selective oxidation of hydro­carbons under mild reaction conditions (Company *et al.*, 2007 ▸). Studies show that highly active non-heme iron catalysts that facilitate efficient stereo-specific alkane hy­droxy­lation using H_2_O_2_ as oxidant can be synthesized by employing tetra­dentate N_4_-donor ligands such as *N*,*N*′-dimethyl-*N*,*N*′-bis­(2-pyridyl­meth­yl)ethane-1,2-di­amine (BPMEN) or *tris*(2-pyridyl­meth­yl)amine (TPMA) (Costas *et al.*, 2000 ▸). These catalysts have provided key insights into possible mechanisms used by enzymes to oxidize alkanes in nature (Meunier *et al.*, 2004 ▸). In addition to the application of four-coordinate iron complexes as catalysts in hy­droxy­lation reactions, studies also show that these complexes can be utilized in epoxidation reactions of terminal and electron-deficient alkenes (Dubois *et al.*, 2003 ▸). Iron oxido-bridging complexes are reported to play an important role in oxygen transport (hemerythrin), phosphate ester hydrolysis (purple acid phosphates), or DNA synthesis (ribonucleotide reductase) (Dutta *et al.*, 1996 ▸). These oxido complexes exhibit redox and magnetic properties making them excellent candidates for future investigations into the mechanisms behind important biological and chemical processes (Feig & Lippard, 1994 ▸). Given the significance and application of iron complexes made from tetra­dentate ligands, herein we report on the synthesis and crystal structure of the solvated title compound [Fe_2_(C_26_H_28_N_4_)(Cl)(*μ*-O)Fe(Cl)_3_]·CHCl_3_ (**1**), incorporating (1*R*,2*R*)-*N*,*N*′-bis­[(quino­lin-2-yl)methyl]cyclo­hexane-1,2-di­amine (Fig. 1 ▸).
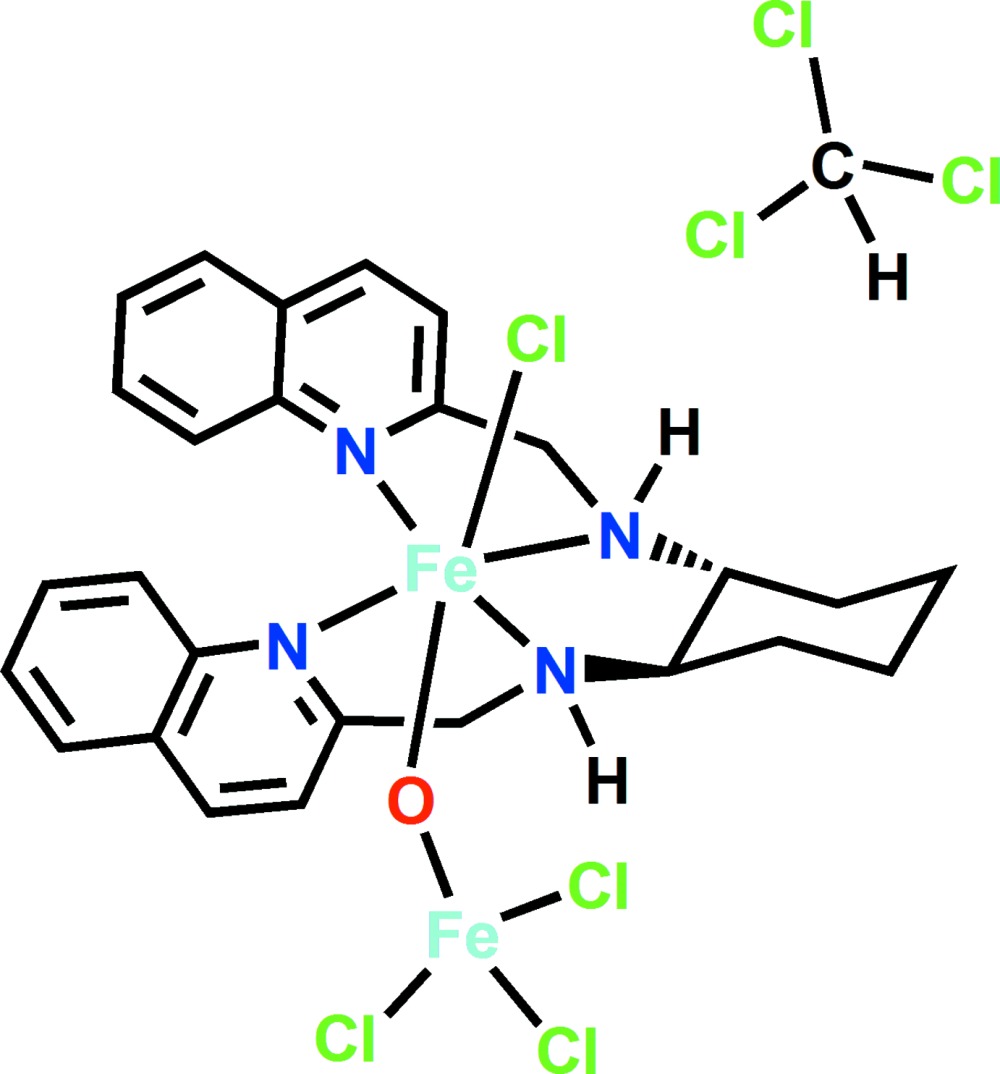


## Structural commentary   

There is one coordination complex and one mol­ecule of chloro­form solvent in the asymmetric unit. The coordination complex features two Fe(III) metal cations. One of the metal cations, Fe1, assumes a distorted octa­hedral coordination (Table 1 ▸). The tetradentate ligand, (1*R*,2*R*)-*N*,*N*′-bis[(quino­lin-2-yl)methyl­]cyclo­hexane-1,2-di­amine, inter­acts with the Fe^III^ cation in the equatorial plane through the four amine groups. A chloride ion and a bridging oxido ligand, which connects the two metal cations, complete the axial coordination. The distortions from the ideal octa­hedral geometry occur both in the equatorial and the axial positions. The equatorial angles vary widely from 74.96 (9)°, as in the case of the N1—Fe1—N2 angle, to 133.98 (9)° for the untethered N1—Fe1—N4 angle. The axial ligands exhibit a bent conformation with a Cl1—Fe1—O1 angle of 166.06 (7)°. In contrast, the second Fe metal cation, Fe2, exhibits a near ideal tetra­hedral coordination geometry composed of one O atom and three Cl atoms. As expected based on the difference in the saturation of the coordination sphere, the Fe—Cl and the Fe—O distances for Fe1 are longer than that for Fe2. The single Fe—Cl distance for Fe1 is 2.3560 (8) Å, whereas the average Fe2—Cl distance of 2.232 (9) Å is more than 0.1 Å shorter, a statistically significant variation. Similarly, the Fe—O distances for Fe1 and Fe2 are also statistically significantly different at 1.808 (2) Å and 1.756 (2) Å, respectively. The bond lengths in the title compound are comparable to the mean Fe—Cl distances from the CSD for Fe complexes in an octa­hedral coordination [2.33 (7) Å] and a tetra­hedral coordination [2.23 (3) Å]. In contrast, the Fe—O distances for both the octa­hedral and tetra­hedral configurations in the title compound are shorter than the mean distances from CSD [2.01 (9) and 1.87 (13) Å, respectively]. A very weak intra­molecular N3—H3⋯Cl4 hydrogen bond (Table 2 ▸) occurs. Finally, we observe that complex (**1**) is present only as the *M* (left-handed) conformer.

## Supra­molecular features   

**Figure 2:**
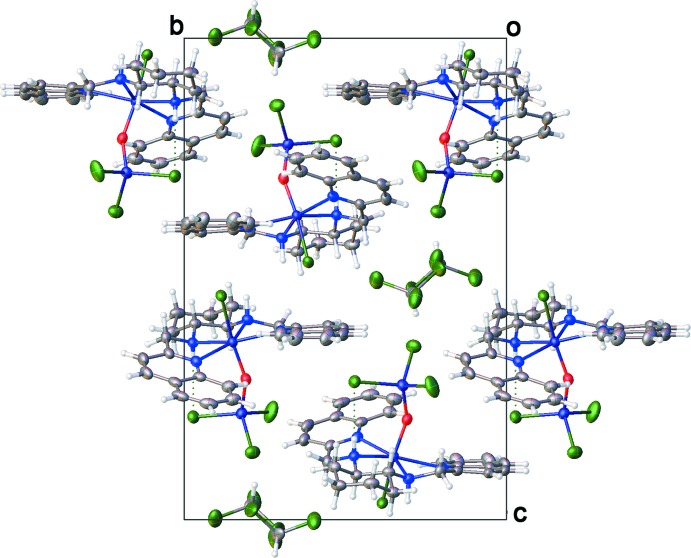
Packing diagram for complex (**1**), showing the columns of the coordination complex and the channels of disordered chloro­form solvent mol­ecules stacked along the *a* axis.

The mol­ecules in the crystal structure are related by twofold screw axes running along the *a*-, *b*-, and *c*-axis directions. As there are no additional symmetry elements present, the resulting space group, *P*2_1_2_1_2_1_, is chiral. The absolute structure was unequivocally established, as evidenced by a Hooft *y* parameter of 0.003 (6), using anomalous dispersion. Apart from a weak C—H⋯Cl bond from the chloro­form mol­ecule to one of the chloride ions bonded to Fe2 (Table 2 ▸), the mol­ecules of the coordination complexes display minimal inter­atomic inter­actions. They assemble into columns that run parallel to the *a* axis. A circular arrangement of six columns of coord­ination complex mol­ecules creates a channel. The channel is filled with solvent chloro­form mol­ecules that exhibit extensive positional disorder. For two of the columns that frame the chloro­form channels, the oxido-trichloride groups of the coordination complexes point into the channels, while the other four columns face the void with the (1*R*,2*R*)-*N*,*N*′-bis­[(quin­olin-2-yl)methyl­]cyclo­hexane-1,2-di­amine lig­and. The packing is illustrated in Fig. 2 ▸.

## Database survey   

In our survey of the Cambridge Structural Database (Groom *et al.*, 2016 ▸), we found five reported structures incorporating the (1*R*,2*R*)-*N*,*N*′-bis­[(quinolin-2-yl)methyl­]cyclo­hex­ane-1,2-di­amine ligand motif. Of the five, only one structure showed coordination to iron (Dengler *et al.*, 2011 ▸). In that structure, the distorted octa­hedral coordination of the Fe^III^ metal atom is completed by two chloride ligands in the axial positions. The two Fe—Cl distances are comparable (2.495 and 2.509 Å) and the Cl1—Fe—N angles show a narrow distribution from 92–94°, except for Cl1—Fe—N1, which is 84°.

## Synthesis and crystallization   

**Figure 3:**
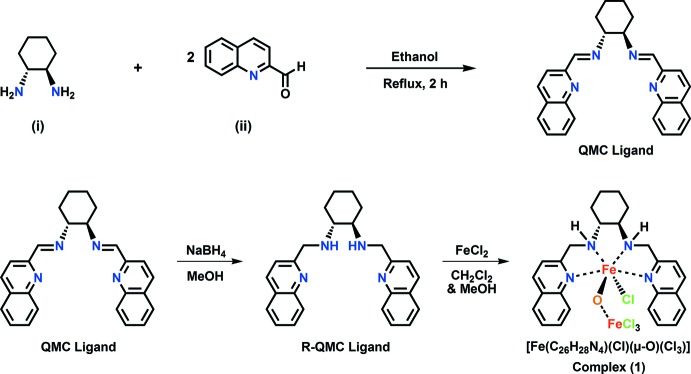
Synthetic scheme for complex (**1**).

**Synthesis of (1** ***R*** **,2** ***R*** **)-** ***N***,***N*** **′-bis­[(quinolin-2-yl)methyl]cyclo­hexane-1,2-di­amine (R-QMC):** In a 50 mL round-bottom flask (1*R*,2*R*)-1,2-cyclo­hexa­nedi­amine (0.20 g, 1.8 mmol) and 2-quinoline­carboxaldehyde (0.55 g, 3.6 mmol) were refluxed in ethanol (10 mL) for 3 h. A yellow precipitate formed that was isolated by filtration, washed twice with ethanol, and dried *in vacuo* producing the unreduced form of the ligand (QMC), (0.63 g, 89% yield). ^1^H NMR (CDCl_3_, 400 MHz): δ 1.24 (*br*, 2 H, CH), 1.56 (*br*, 2 H, CH), 1.97 (*br*, 2 H, CH), 3.62 (*br*, 2 H, CH), 7.48 (*t*, 1 H, *J* = 8.06 Hz, CH), 7.65 (*t*, 1 H, *J* = 8.06 Hz, CH), 7.74 (*d*, 1 H, *J* = 8.06 Hz, CH), 8.03 (*d*, 1 H, *J* = 8.56 Hz, CH), 8.06 (*s*, 1 H, CH), and 8.52 (*s*, 1 H, CH). The reduced form of the ligand (R-QMC, Fig. 3 ▸) was synthesized by reacting ligand QMC (0.50 g, 1.3 mmol) with sodium borohydride (0.06 g, 1.5 mmol) in methanol at room temperature for 12 h to produce R-QMC (0.42 g, 82% yield). ^1^H NMR (CDCl_3_, 400 MHz): δ 1.24 (*br*, 2 H, CH), 1.56 (*br*, 2 H, CH), 1.97 (*br*, 2 H, CH), 3.62 (*br*, 2 H, CH), 4.22 (*dd*, 2 H, *J* = 8.06 Hz, CH), 7.55 (*t*, 1 H, *J* = 8.06 Hz, CH), 7.61–7.73 (*m*, CH), 7.81 (*d*, 1 H, *J* = 8.56 Hz, CH), 8.06 (*d*, 1 H *J* = 8.06 Hz, CH), 8.08 (*d*, 1 H *J* = 8.06 Hz, CH).

**Synthesis of ({(1*R*,2*R*)-*N*,*N*′-bis­[(quinolin-2-yl)methyl]cyclo­hexane-1,2-di­amine}­chlorido­iron(III))-μ-oxido-[tri­chlo­rido­ferrate(III)] chloro­form monosolvate** R-QMC (0.25 g, 0.63 mmol) was dissolved in 50/50 mixture of di­chloro­methane and ethanol (20 mL) in a 50 mL round-bottom flask. Iron(II) chloride (0.08 g, 0.63 mmol) was added to the flask to give a brown-colored solution. The reaction was allowed to mix for 6 h under gentle heat producing a brown-colored precipitate. The precipitate was filtered and washed twice with cold solvent then dried under vacuum for 30 minutes producing a brown powder (0.19 g, 58%). Brown prisms of (**1**) suitable for X-ray analysis were obtained by slow solvent diffusion of diethyl ether into a concentrated complex solution made in chloro­form.

## Refinement   

Crystal data, data collection and structure refinement details are summarized in Table 3 ▸. All hydrogen atoms, except for the amine hydrogen atom bonded to N3, were added at idealized positions and were allowed to ride on the neighboring atoms with relative isotropic displacement coefficients. The amine hydrogen bonded to N3 was allowed to refine freely. In addition to the {[(1*R*,2*R*)-*N*,*N*′-bis­[(quinolin-2-yl)methyl]cyclo­hexane-1,2-di­amine]­chlorido­iron(III)}-μ-oxido-[tri­chlo­rido­ferrate(III)], there is one mol­ecule of chloro­form solvent in the asymmetric unit. The solvent mol­ecule exhibits extensive positional disorder over three positions. Initially, the disorder was modeled with chloro­form mol­ecule in an idealized geometry, where the 1,2 and the 1,3 bond lengths were constrained. As the refinement converged, the geometry constraints were lifted. The chlorine atoms Cl6 and Cl7 were modeled over two positions, with the major component contributing 54.4 (3)%. The carbon atom C27 required modeling over three positions with the major component contribution of 54.4 (3)% and the two minor components contributing 24.1 (4)% and 21.5 (4)%. The C–Cl distances for all of the disorder components were restrained to be similar. In addition, Cl6*A*–Cl7*A* and Cl7*A*–Cl5 were restrained to be similar. The absolute structure was unequivocally determined by anomalous dispersion.

## Supplementary Material

Crystal structure: contains datablock(s) I. DOI: 10.1107/S2056989017007952/hb7682sup1.cif

Structure factors: contains datablock(s) I. DOI: 10.1107/S2056989017007952/hb7682Isup2.hkl

CCDC reference: 1552964

Additional supporting information:  crystallographic information; 3D view; checkCIF report

## Figures and Tables

**Table 1:** Selected bond lengths (Å)

Fe1—O1	1.808 (2)	Fe1—Cl1	2.3560 (8)
Fe1—N1	2.243 (2)	Fe2—O1	1.756 (2)
Fe1—N2	2.172 (3)	Fe2—Cl2	2.2194 (9)
Fe1—N3	2.159 (2)	Fe2—Cl3	2.2331 (10)
Fe1—N4	2.223 (2)	Fe2—Cl4	2.2432 (9)

**Table 2:** Hydrogen-bond geometry (Å, °)

*D*—H⋯*A*	*D*—H	H⋯*A*	*D*⋯*A*	*D*—H⋯*A*
N3—H3⋯Cl4	0.80 (4)	2.60 (4)	3.378 (3)	167 (3)
C27*A*—H27*A*⋯Cl2^i^	1.00	2.41	3.30 (2)	149

Symmetry code: (i) 
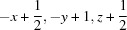
.

**Table 3:** Experimental details

Crystal data	
Chemical formula	[Fe_2_Cl_4_O(C_26_H_28_N_4_)]·CHCl_3_
*M*_r_	785.39
Crystal system, space group	Orthorhombic, *P*2_1_2_1_2_1_
Temperature (K)	120
*a*, *b*, *c* (Å)	10.3489 (6), 14.3664 (8), 21.4619 (13)
*V* (Å^3^)	3190.9 (3)
*Z*	4
Radiation type	Mo *K*α
μ (mm^−1^)	1.53
Crystal size (mm)	0.26 × 0.22 × 0.14

Data collection	
Diffractometer	Bruker APEXII CCD
Absorption correction	Multi-scan (*SADABS*; Bruker, 2016 ▸)
*T*_min_, *T*_max_	0.658, 0.746
No. of measured, independent and observed [*I* > 2σ(*I*)] reflections	79886, 10537, 8808
*R*_int_	0.065
(sin θ/λ)_max_ (Å^−1^)	0.736

Refinement	
*R*[*F* ^2^ > 2σ(*F* ^2^)], *wR*(*F* ^2^), *S*	0.037, 0.082, 1.05
No. of reflections	10537
No. of parameters	413
No. of restraints	37
H-atom treatment	H atoms treated by a mixture of independent and constrained refinement
Δρ_max_, Δρ_min_ (e Å^−3^)	0.67, −0.52
Absolute structure parameter	0.000 (14)

Computer programs: *APEX2* and *SAINT* (Bruker, 2016 ▸), *SHELXT* (Sheldrick, 2015*a* ▸), *SHELXL2014* (Sheldrick, 2015*b* ▸) and *OLEX2* (Dolomanov *et al.*, 2009 ▸).

## supplementary crystallographic information

### Crystal data

**Table:**

[Fe_2_Cl_4_O(C_26_H_28_N_4_)]·CHCl_3_	*D*_x_ = 1.635 Mg m^−^^3^
*M**_r_* = 785.39	Mo *K*α radiation, λ = 0.71073 Å
Orthorhombic, *P*2_1_2_1_2_1_	Cell parameters from 9981 reflections
*a* = 10.3489 (6) Å	θ = 2.7–30.8°
*b* = 14.3664 (8) Å	µ = 1.53 mm^−^^1^
*c* = 21.4619 (13) Å	*T* = 120 K
*V* = 3190.9 (3) Å^3^	Prism, brown
*Z* = 4	0.26 × 0.22 × 0.14 mm
*F*(000) = 1592	

### Data collection

**Table:**

Bruker APEXII CCD diffractometer	8808 reflections with *I* > 2σ(*I*)
φ and ω scans	*R*_int_ = 0.065
Absorption correction: multi-scan (SADABS; Bruker, 2016)	θ_max_ = 31.6°, θ_min_ = 2.4°
*T*_min_ = 0.658, *T*_max_ = 0.746	*h* = −14→15
79886 measured reflections	*k* = −20→20
10537 independent reflections	*l* = −30→31

### Refinement

**Table:**

Refinement on *F*^2^	Hydrogen site location: mixed
Least-squares matrix: full	H atoms treated by a mixture of independent and constrained refinement
*R*[*F*^2^ > 2σ(*F*^2^)] = 0.037	*w* = 1/[σ^2^(*F*_o_^2^) + (0.0361*P*)^2^ + 0.5065*P*] where *P* = (*F*_o_^2^ + 2*F*_c_^2^)/3
*wR*(*F*^2^) = 0.082	(Δ/σ)_max_ = 0.001
*S* = 1.05	Δρ_max_ = 0.67 e Å^−^^3^
10537 reflections	Δρ_min_ = −0.52 e Å^−^^3^
413 parameters	Absolute structure: Refined as an inversion twin
37 restraints	Absolute structure parameter: 0.000 (14)
Primary atom site location: dual	

### Special details

**Table:**

Geometry. All esds (except the esd in the dihedral angle between two l.s. planes) are estimated using the full covariance matrix. The cell esds are taken into account individually in the estimation of esds in distances, angles and torsion angles; correlations between esds in cell parameters are only used when they are defined by crystal symmetry. An approximate (isotropic) treatment of cell esds is used for estimating esds involving l.s. planes.
Refinement. Refined as a 2-component inversion twin.

### Fractional atomic coordinates and isotropic or equivalent isotropic displacement parameters (Å^2^)

**Table:**

	*x*	*y*	*z*	*U*_iso_*/*U*_eq_	Occ. (<1)
Fe1	0.24940 (4)	0.34973 (3)	0.86909 (2)	0.02294 (9)	
Fe2	0.37047 (4)	0.32209 (3)	0.72075 (2)	0.02735 (10)	
Cl1	0.15040 (7)	0.38505 (5)	0.96507 (4)	0.03021 (16)	
Cl2	0.24573 (9)	0.28798 (6)	0.63979 (4)	0.04158 (19)	
Cl3	0.54491 (9)	0.23071 (7)	0.72381 (7)	0.0617 (3)	
Cl4	0.43247 (8)	0.47130 (5)	0.71335 (4)	0.03348 (16)	
O1	0.28867 (19)	0.30834 (14)	0.79183 (10)	0.0295 (5)	
N1	0.2138 (2)	0.19951 (16)	0.89191 (12)	0.0257 (5)	
N2	0.4247 (2)	0.30306 (17)	0.91515 (12)	0.0276 (5)	
H2	0.4088	0.3066	0.9611	0.033*	
N3	0.3688 (2)	0.47300 (16)	0.86772 (13)	0.0244 (5)	
H3	0.397 (3)	0.472 (2)	0.8331 (18)	0.029*	
N4	0.1286 (2)	0.46258 (17)	0.82871 (12)	0.0259 (5)	
C1	0.0979 (3)	0.1519 (2)	0.88928 (14)	0.0279 (6)	
C2	−0.0189 (3)	0.2008 (2)	0.88477 (17)	0.0357 (7)	
H2A	−0.0188	0.2669	0.8860	0.043*	
C3	−0.1336 (3)	0.1535 (3)	0.8786 (2)	0.0474 (9)	
H3A	−0.2119	0.1875	0.8746	0.057*	
C4	−0.1369 (4)	0.0557 (3)	0.8781 (2)	0.0492 (10)	
H4	−0.2167	0.0239	0.8733	0.059*	
C5	−0.0246 (4)	0.0067 (2)	0.88457 (17)	0.0415 (9)	
H5	−0.0269	−0.0594	0.8849	0.050*	
C6	0.0945 (3)	0.0530 (2)	0.89077 (15)	0.0319 (7)	
C7	0.2130 (4)	0.0055 (2)	0.89797 (15)	0.0339 (7)	
H7	0.2143	−0.0606	0.8990	0.041*	
C8	0.3249 (3)	0.0539 (2)	0.90334 (15)	0.0318 (7)	
H8	0.4042	0.0221	0.9101	0.038*	
C9	0.3225 (3)	0.1519 (2)	0.89887 (14)	0.0273 (6)	
C10	0.4472 (3)	0.2049 (2)	0.89977 (17)	0.0320 (7)	
H10A	0.5061	0.1770	0.9310	0.038*	
H10B	0.4892	0.2004	0.8584	0.038*	
C11	0.5348 (3)	0.3655 (2)	0.90109 (15)	0.0266 (6)	
H11	0.5616	0.3551	0.8569	0.032*	
C12	0.6531 (3)	0.3515 (2)	0.94307 (15)	0.0314 (6)	
H12A	0.6286	0.3619	0.9871	0.038*	
H12B	0.6848	0.2867	0.9390	0.038*	
C13	0.7603 (3)	0.4192 (2)	0.92475 (16)	0.0367 (7)	
H13A	0.7889	0.4058	0.8817	0.044*	
H13B	0.8353	0.4106	0.9528	0.044*	
C14	0.7134 (3)	0.5200 (3)	0.92877 (16)	0.0369 (8)	
H14A	0.6956	0.5360	0.9728	0.044*	
H14B	0.7822	0.5622	0.9135	0.044*	
C15	0.5912 (3)	0.5347 (2)	0.89019 (16)	0.0316 (7)	
H15A	0.5587	0.5988	0.8970	0.038*	
H15B	0.6123	0.5281	0.8454	0.038*	
C16	0.4860 (3)	0.4654 (2)	0.90736 (14)	0.0260 (6)	
H16	0.4605	0.4762	0.9517	0.031*	
C17	0.2910 (3)	0.5562 (2)	0.87921 (16)	0.0296 (6)	
H17A	0.3375	0.6120	0.8641	0.036*	
H17B	0.2756	0.5634	0.9245	0.036*	
C18	0.1644 (3)	0.5469 (2)	0.84567 (15)	0.0288 (6)	
C19	0.0884 (3)	0.6267 (2)	0.83377 (17)	0.0374 (8)	
H19	0.1182	0.6866	0.8458	0.045*	
C20	−0.0278 (4)	0.6169 (2)	0.80486 (17)	0.0390 (8)	
H20	−0.0808	0.6699	0.7977	0.047*	
C21	−0.0696 (3)	0.5276 (2)	0.78549 (15)	0.0327 (7)	
C22	−0.1905 (3)	0.5116 (3)	0.75714 (16)	0.0400 (9)	
H22	−0.2471	0.5624	0.7494	0.048*	
C23	−0.2267 (3)	0.4237 (3)	0.74077 (16)	0.0420 (9)	
H23	−0.3098	0.4132	0.7233	0.050*	
C24	−0.1422 (3)	0.3490 (3)	0.74965 (16)	0.0398 (8)	
H24	−0.1675	0.2883	0.7370	0.048*	
C25	−0.0227 (3)	0.3622 (2)	0.77650 (16)	0.0327 (7)	
H	0.0349	0.3112	0.7811	0.039*	
C26	0.0136 (3)	0.4510 (2)	0.79695 (14)	0.0276 (6)	
Cl5	0.04556 (12)	0.59674 (7)	1.00240 (6)	0.0613 (3)	
Cl6	0.0633 (4)	0.79088 (18)	0.97904 (15)	0.0776 (11)	0.544 (3)
Cl6A	−0.0063 (4)	0.7908 (4)	0.9720 (2)	0.0927 (17)	0.456 (3)
Cl7	−0.1718 (2)	0.71764 (14)	1.04018 (13)	0.0676 (7)	0.544 (3)
Cl7A	−0.0951 (2)	0.70539 (15)	1.08266 (12)	0.0531 (7)	0.456 (3)
C27	−0.0048 (11)	0.7090 (7)	1.0301 (6)	0.046 (4)	0.544 (3)
H27	0.0360	0.7186	1.0718	0.055*	0.544 (3)
C27A	0.030 (2)	0.7114 (11)	1.0304 (9)	0.027 (5)	0.241 (4)
H27A	0.1111	0.7305	1.0522	0.032*	0.241 (4)
C27B	−0.0529 (16)	0.6944 (8)	1.0038 (7)	0.042 (5)	0.215 (4)
H27B	−0.1340	0.6761	0.9816	0.050*	0.215 (4)

### Atomic displacement parameters (Å^2^)

**Table:**

	*U*^11^	*U*^22^	*U*^33^	*U*^12^	*U*^13^	*U*^23^
Fe1	0.02119 (17)	0.02163 (17)	0.0260 (2)	0.00049 (15)	0.00144 (18)	0.00237 (15)
Fe2	0.0243 (2)	0.0275 (2)	0.0302 (2)	0.00048 (16)	0.00332 (18)	−0.00318 (17)
Cl1	0.0337 (4)	0.0278 (3)	0.0291 (4)	0.0003 (3)	0.0073 (3)	0.0025 (3)
Cl2	0.0377 (4)	0.0501 (5)	0.0369 (4)	−0.0118 (4)	−0.0030 (4)	−0.0031 (3)
Cl3	0.0351 (4)	0.0454 (5)	0.1045 (9)	0.0137 (4)	0.0018 (5)	−0.0252 (6)
Cl4	0.0340 (4)	0.0311 (3)	0.0354 (4)	−0.0051 (3)	0.0032 (3)	0.0020 (3)
O1	0.0321 (11)	0.0242 (10)	0.0323 (12)	0.0011 (8)	0.0013 (9)	0.0037 (8)
N1	0.0286 (12)	0.0220 (11)	0.0265 (13)	0.0005 (9)	0.0003 (10)	0.0022 (9)
N2	0.0271 (12)	0.0298 (13)	0.0260 (13)	−0.0001 (10)	−0.0009 (10)	0.0025 (10)
N3	0.0245 (12)	0.0255 (11)	0.0231 (12)	−0.0012 (9)	0.0020 (11)	0.0015 (10)
N4	0.0223 (12)	0.0284 (12)	0.0271 (13)	0.0042 (10)	0.0030 (10)	0.0029 (10)
C1	0.0326 (15)	0.0273 (14)	0.0237 (14)	−0.0049 (11)	0.0017 (12)	0.0018 (11)
C2	0.0304 (16)	0.0346 (16)	0.042 (2)	−0.0018 (12)	0.0051 (14)	−0.0025 (14)
C3	0.0321 (17)	0.048 (2)	0.062 (3)	−0.0058 (15)	0.0095 (17)	−0.0116 (19)
C4	0.0396 (19)	0.048 (2)	0.060 (3)	−0.0161 (16)	0.0080 (19)	−0.0117 (18)
C5	0.056 (2)	0.0312 (16)	0.038 (2)	−0.0146 (15)	0.0046 (17)	−0.0048 (14)
C6	0.0449 (19)	0.0266 (14)	0.0242 (15)	−0.0042 (12)	0.0031 (14)	−0.0008 (12)
C7	0.054 (2)	0.0233 (14)	0.0246 (15)	0.0014 (13)	0.0005 (14)	0.0018 (12)
C8	0.0449 (19)	0.0279 (14)	0.0225 (15)	0.0067 (12)	−0.0027 (13)	0.0009 (12)
C9	0.0332 (15)	0.0276 (13)	0.0210 (14)	0.0043 (12)	−0.0020 (12)	0.0028 (11)
C10	0.0294 (15)	0.0298 (15)	0.0368 (18)	0.0054 (12)	−0.0052 (14)	0.0021 (13)
C11	0.0205 (13)	0.0353 (15)	0.0240 (15)	−0.0015 (11)	−0.0002 (11)	−0.0001 (12)
C12	0.0264 (14)	0.0416 (16)	0.0261 (15)	0.0003 (13)	0.0003 (12)	−0.0006 (13)
C13	0.0272 (15)	0.054 (2)	0.0284 (16)	−0.0016 (15)	−0.0033 (14)	0.0001 (14)
C14	0.0324 (16)	0.053 (2)	0.0256 (16)	−0.0133 (14)	−0.0024 (13)	−0.0007 (14)
C15	0.0283 (15)	0.0386 (16)	0.0277 (16)	−0.0086 (12)	0.0020 (13)	−0.0011 (13)
C16	0.0247 (14)	0.0310 (14)	0.0223 (15)	−0.0041 (11)	0.0006 (11)	0.0011 (11)
C17	0.0318 (15)	0.0230 (13)	0.0339 (18)	−0.0018 (11)	0.0038 (13)	0.0010 (12)
C18	0.0319 (15)	0.0270 (14)	0.0275 (16)	0.0040 (11)	0.0070 (13)	0.0058 (11)
C19	0.047 (2)	0.0276 (16)	0.0380 (19)	0.0091 (13)	0.0060 (16)	0.0046 (13)
C20	0.044 (2)	0.0367 (17)	0.0366 (19)	0.0175 (15)	0.0084 (15)	0.0111 (14)
C21	0.0302 (15)	0.0430 (17)	0.0250 (16)	0.0113 (13)	0.0077 (13)	0.0096 (14)
C22	0.0293 (16)	0.065 (2)	0.0262 (17)	0.0171 (16)	0.0047 (13)	0.0148 (16)
C23	0.0234 (17)	0.074 (3)	0.0284 (17)	0.0031 (15)	−0.0004 (13)	0.0066 (17)
C24	0.0303 (16)	0.057 (2)	0.0321 (18)	−0.0008 (16)	0.0002 (14)	−0.0039 (15)
C25	0.0257 (14)	0.0415 (17)	0.0308 (17)	0.0046 (12)	0.0004 (13)	−0.0028 (14)
C26	0.0237 (14)	0.0374 (15)	0.0216 (15)	0.0060 (11)	0.0036 (11)	0.0046 (12)
Cl5	0.0708 (7)	0.0439 (5)	0.0691 (7)	−0.0069 (5)	0.0295 (6)	0.0027 (5)
Cl6	0.143 (3)	0.0404 (12)	0.0489 (14)	−0.0417 (17)	0.011 (2)	0.0081 (10)
Cl6A	0.096 (3)	0.122 (3)	0.060 (2)	0.051 (3)	0.010 (2)	0.042 (2)
Cl7	0.0658 (13)	0.0416 (10)	0.0953 (19)	0.0037 (9)	−0.0259 (13)	−0.0139 (10)
Cl7A	0.0577 (14)	0.0441 (11)	0.0573 (15)	−0.0032 (9)	0.0153 (11)	−0.0198 (10)
C27	0.059 (9)	0.043 (5)	0.036 (6)	−0.033 (5)	−0.012 (5)	0.001 (4)
C27A	0.023 (9)	0.038 (10)	0.019 (9)	−0.011 (6)	−0.010 (6)	0.000 (7)
C27B	0.025 (8)	0.027 (8)	0.073 (15)	−0.010 (6)	−0.002 (8)	−0.012 (8)

### Geometric parameters (Å, º)

**Table:**

Fe1—O1	1.808 (2)	C12—H12B	0.9900
Fe1—N1	2.243 (2)	C12—C13	1.527 (5)
Fe1—N2	2.172 (3)	C13—H13A	0.9900
Fe1—N3	2.159 (2)	C13—H13B	0.9900
Fe1—N4	2.223 (2)	C13—C14	1.530 (5)
Fe1—Cl1	2.3560 (8)	C14—H14A	0.9900
Fe2—O1	1.756 (2)	C14—H14B	0.9900
Fe2—Cl2	2.2194 (9)	C14—C15	1.526 (4)
Fe2—Cl3	2.2331 (10)	C15—H15A	0.9900
Fe2—Cl4	2.2432 (9)	C15—H15B	0.9900
N1—C1	1.382 (4)	C15—C16	1.521 (4)
N1—C9	1.324 (4)	C16—H16	1.0000
N2—H2	1.0000	C17—H17A	0.9900
N2—C10	1.467 (4)	C17—H17B	0.9900
N2—C11	1.481 (4)	C17—C18	1.501 (4)
N3—H3	0.80 (4)	C18—C19	1.414 (4)
N3—C16	1.486 (4)	C19—H19	0.9500
N3—C17	1.462 (4)	C19—C20	1.361 (5)
N4—C18	1.318 (4)	C20—H20	0.9500
N4—C26	1.381 (4)	C20—C21	1.417 (5)
C1—C2	1.401 (4)	C21—C22	1.411 (5)
C1—C6	1.422 (4)	C21—C26	1.418 (4)
C2—H2A	0.9500	C22—H22	0.9500
C2—C3	1.374 (5)	C22—C23	1.363 (6)
C3—H3A	0.9500	C23—H23	0.9500
C3—C4	1.405 (5)	C23—C24	1.398 (5)
C4—H4	0.9500	C24—H24	0.9500
C4—C5	1.366 (6)	C24—C25	1.378 (5)
C5—H5	0.9500	C25—H	0.9500
C5—C6	1.407 (5)	C25—C26	1.401 (5)
C6—C7	1.412 (5)	Cl5—C27	1.796 (11)
C7—H7	0.9500	Cl5—C27A	1.761 (17)
C7—C8	1.355 (5)	Cl5—C27B	1.733 (13)
C8—H8	0.9500	Cl6—C27	1.755 (10)
C8—C9	1.413 (4)	Cl6A—C27A	1.735 (17)
C9—C10	1.498 (4)	Cl6A—C27B	1.618 (12)
C10—H10A	0.9900	Cl7—C27	1.746 (11)
C10—H10B	0.9900	Cl7A—C27A	1.716 (17)
C11—H11	1.0000	Cl7A—C27B	1.755 (15)
C11—C12	1.534 (4)	C27—H27	1.0000
C11—C16	1.527 (4)	C27A—H27A	1.0000
C12—H12A	0.9900	C27B—H27B	1.0000
			
O1—Fe1—Cl1	166.06 (7)	C13—C12—C11	110.2 (3)
O1—Fe1—N1	85.45 (9)	C13—C12—H12A	109.6
O1—Fe1—N2	97.36 (10)	C13—C12—H12B	109.6
O1—Fe1—N3	97.40 (10)	C12—C13—H13A	109.4
O1—Fe1—N4	90.50 (9)	C12—C13—H13B	109.4
N1—Fe1—Cl1	86.84 (7)	C12—C13—C14	111.0 (3)
N2—Fe1—Cl1	91.81 (7)	H13A—C13—H13B	108.0
N2—Fe1—N1	74.96 (9)	C14—C13—H13A	109.4
N2—Fe1—N4	150.72 (10)	C14—C13—H13B	109.4
N3—Fe1—Cl1	94.82 (7)	C13—C14—H14A	109.4
N3—Fe1—N1	152.34 (9)	C13—C14—H14B	109.4
N3—Fe1—N2	77.39 (9)	H14A—C14—H14B	108.0
N3—Fe1—N4	73.64 (9)	C15—C14—C13	111.3 (3)
N4—Fe1—Cl1	86.52 (7)	C15—C14—H14A	109.4
N4—Fe1—N1	133.98 (9)	C15—C14—H14B	109.4
Cl2—Fe2—Cl3	111.31 (4)	C14—C15—H15A	109.3
Cl2—Fe2—Cl4	108.78 (4)	C14—C15—H15B	109.3
Cl3—Fe2—Cl4	109.42 (4)	H15A—C15—H15B	107.9
O1—Fe2—Cl2	112.02 (8)	C16—C15—C14	111.8 (3)
O1—Fe2—Cl3	107.35 (8)	C16—C15—H15A	109.3
O1—Fe2—Cl4	107.87 (7)	C16—C15—H15B	109.3
Fe2—O1—Fe1	150.26 (13)	N3—C16—C11	106.8 (2)
C1—N1—Fe1	127.54 (19)	N3—C16—C15	113.4 (2)
C9—N1—Fe1	112.45 (19)	N3—C16—H16	108.5
C9—N1—C1	119.1 (3)	C11—C16—H16	108.5
Fe1—N2—H2	107.2	C15—C16—C11	111.0 (3)
C10—N2—Fe1	109.10 (19)	C15—C16—H16	108.5
C10—N2—H2	107.2	N3—C17—H17A	109.9
C10—N2—C11	114.5 (2)	N3—C17—H17B	109.9
C11—N2—Fe1	111.27 (18)	N3—C17—C18	109.1 (2)
C11—N2—H2	107.2	H17A—C17—H17B	108.3
Fe1—N3—H3	102 (3)	C18—C17—H17A	109.9
C16—N3—Fe1	113.52 (17)	C18—C17—H17B	109.9
C16—N3—H3	104 (3)	N4—C18—C17	117.4 (3)
C17—N3—Fe1	110.66 (17)	N4—C18—C19	122.7 (3)
C17—N3—H3	112 (3)	C19—C18—C17	120.0 (3)
C17—N3—C16	114.4 (2)	C18—C19—H19	120.3
C18—N4—Fe1	113.9 (2)	C20—C19—C18	119.3 (3)
C18—N4—C26	119.3 (3)	C20—C19—H19	120.3
C26—N4—Fe1	126.1 (2)	C19—C20—H20	120.1
N1—C1—C2	120.3 (3)	C19—C20—C21	119.8 (3)
N1—C1—C6	121.0 (3)	C21—C20—H20	120.1
C2—C1—C6	118.7 (3)	C20—C21—C26	117.8 (3)
C1—C2—H2A	119.8	C22—C21—C20	123.0 (3)
C3—C2—C1	120.3 (3)	C22—C21—C26	119.2 (3)
C3—C2—H2A	119.8	C21—C22—H22	119.8
C2—C3—H3A	119.5	C23—C22—C21	120.4 (3)
C2—C3—C4	121.0 (4)	C23—C22—H22	119.8
C4—C3—H3A	119.5	C22—C23—H23	119.9
C3—C4—H4	120.2	C22—C23—C24	120.3 (3)
C5—C4—C3	119.6 (3)	C24—C23—H23	119.9
C5—C4—H4	120.2	C23—C24—H24	119.6
C4—C5—H5	119.6	C25—C24—C23	120.9 (4)
C4—C5—C6	120.8 (3)	C25—C24—H24	119.6
C6—C5—H5	119.6	C24—C25—H	120.1
C5—C6—C1	119.5 (3)	C24—C25—C26	119.8 (3)
C5—C6—C7	122.9 (3)	C26—C25—H	120.1
C7—C6—C1	117.7 (3)	N4—C26—C21	121.0 (3)
C6—C7—H7	119.9	N4—C26—C25	119.7 (3)
C8—C7—C6	120.2 (3)	C25—C26—C21	119.3 (3)
C8—C7—H7	119.9	C27B—Cl5—C27A	35.2 (10)
C7—C8—H8	120.3	C27B—Cl6A—C27A	36.6 (11)
C7—C8—C9	119.4 (3)	C27A—Cl7A—C27B	35.5 (11)
C9—C8—H8	120.3	Cl5—C27—H27	107.3
N1—C9—C8	122.5 (3)	Cl6—C27—Cl5	106.2 (6)
N1—C9—C10	118.1 (3)	Cl6—C27—H27	107.3
C8—C9—C10	119.4 (3)	Cl7—C27—Cl5	113.1 (7)
N2—C10—C9	110.7 (3)	Cl7—C27—Cl6	115.3 (7)
N2—C10—H10A	109.5	Cl7—C27—H27	107.3
N2—C10—H10B	109.5	Cl5—C27A—H27A	109.9
C9—C10—H10A	109.5	Cl6A—C27A—Cl5	112.9 (11)
C9—C10—H10B	109.5	Cl6A—C27A—H27A	109.9
H10A—C10—H10B	108.1	Cl7A—C27A—Cl5	104.2 (9)
N2—C11—H11	108.4	Cl7A—C27A—Cl6A	110.0 (11)
N2—C11—C12	114.5 (2)	Cl7A—C27A—H27A	109.9
N2—C11—C16	107.3 (2)	Cl5—C27B—Cl7A	103.7 (8)
C12—C11—H11	108.4	Cl5—C27B—H27B	105.9
C16—C11—H11	108.4	Cl6A—C27B—Cl5	120.7 (8)
C16—C11—C12	109.6 (3)	Cl6A—C27B—Cl7A	113.8 (8)
C11—C12—H12A	109.6	Cl6A—C27B—H27B	105.9
C11—C12—H12B	109.6	Cl7A—C27B—H27B	105.9
H12A—C12—H12B	108.1		
			
Fe1—N1—C1—C2	−14.7 (4)	C9—N1—C1—C2	177.1 (3)
Fe1—N1—C1—C6	165.0 (2)	C9—N1—C1—C6	−3.2 (4)
Fe1—N1—C9—C8	−169.5 (2)	C10—N2—C11—C12	−69.3 (4)
Fe1—N1—C9—C10	8.4 (3)	C10—N2—C11—C16	168.8 (3)
Fe1—N2—C10—C9	−39.2 (3)	C11—N2—C10—C9	−164.6 (3)
Fe1—N2—C11—C12	166.5 (2)	C11—C12—C13—C14	57.7 (4)
Fe1—N2—C11—C16	44.6 (3)	C12—C11—C16—N3	−177.7 (2)
Fe1—N3—C16—C11	37.6 (3)	C12—C11—C16—C15	58.2 (3)
Fe1—N3—C16—C15	160.1 (2)	C12—C13—C14—C15	−54.6 (4)
Fe1—N3—C17—C18	−39.6 (3)	C13—C14—C15—C16	53.5 (4)
Fe1—N4—C18—C17	10.4 (3)	C14—C15—C16—N3	−175.9 (3)
Fe1—N4—C18—C19	−169.2 (2)	C14—C15—C16—C11	−55.7 (4)
Fe1—N4—C26—C21	165.7 (2)	C16—N3—C17—C18	−169.4 (2)
Fe1—N4—C26—C25	−13.3 (4)	C16—C11—C12—C13	−59.2 (3)
Cl1—Fe1—O1—Fe2	149.00 (17)	C17—N3—C16—C11	165.9 (2)
Cl2—Fe2—O1—Fe1	−136.6 (2)	C17—N3—C16—C15	−71.5 (3)
Cl3—Fe2—O1—Fe1	100.9 (2)	C17—C18—C19—C20	−178.2 (3)
Cl4—Fe2—O1—Fe1	−16.9 (3)	C18—N4—C26—C21	−3.6 (4)
N1—Fe1—O1—Fe2	−154.4 (3)	C18—N4—C26—C25	177.4 (3)
N1—C1—C2—C3	176.3 (3)	C18—C19—C20—C21	−1.8 (5)
N1—C1—C6—C5	−176.5 (3)	C19—C20—C21—C22	177.8 (3)
N1—C1—C6—C7	2.9 (5)	C19—C20—C21—C26	−0.4 (5)
N1—C9—C10—N2	20.6 (4)	C20—C21—C22—C23	−178.5 (3)
N2—Fe1—O1—Fe2	−80.2 (3)	C20—C21—C26—N4	3.2 (4)
N2—C11—C12—C13	−179.8 (3)	C20—C21—C26—C25	−177.8 (3)
N2—C11—C16—N3	−52.8 (3)	C21—C22—C23—C24	−2.6 (5)
N2—C11—C16—C15	−176.9 (2)	C22—C21—C26—N4	−175.2 (3)
N3—Fe1—O1—Fe2	−2.1 (3)	C22—C21—C26—C25	3.8 (4)
N3—C17—C18—N4	19.0 (4)	C22—C23—C24—C25	1.8 (5)
N3—C17—C18—C19	−161.3 (3)	C23—C24—C25—C26	1.9 (5)
N4—Fe1—O1—Fe2	71.5 (3)	C24—C25—C26—N4	174.4 (3)
N4—C18—C19—C20	1.4 (5)	C24—C25—C26—C21	−4.6 (5)
C1—N1—C9—C8	0.4 (5)	C26—N4—C18—C17	−179.1 (3)
C1—N1—C9—C10	178.3 (3)	C26—N4—C18—C19	1.3 (4)
C1—C2—C3—C4	1.5 (6)	C26—C21—C22—C23	−0.2 (5)
C1—C6—C7—C8	0.2 (5)	C27A—Cl5—C27B—Cl6A	64.6 (14)
C2—C1—C6—C5	3.2 (5)	C27A—Cl5—C27B—Cl7A	−64.3 (12)
C2—C1—C6—C7	−177.4 (3)	C27A—Cl6A—C27B—Cl5	−62.5 (14)
C2—C3—C4—C5	0.8 (6)	C27A—Cl6A—C27B—Cl7A	61.7 (12)
C3—C4—C5—C6	−1.0 (6)	C27A—Cl7A—C27B—Cl5	66.9 (12)
C4—C5—C6—C1	−1.0 (5)	C27A—Cl7A—C27B—Cl6A	−66.1 (12)
C4—C5—C6—C7	179.6 (4)	C27B—Cl5—C27A—Cl6A	−51.8 (12)
C5—C6—C7—C8	179.6 (3)	C27B—Cl5—C27A—Cl7A	67.5 (14)
C6—C1—C2—C3	−3.4 (5)	C27B—Cl6A—C27A—Cl5	54.6 (12)
C6—C7—C8—C9	−2.8 (5)	C27B—Cl6A—C27A—Cl7A	−61.3 (14)
C7—C8—C9—N1	2.6 (5)	C27B—Cl7A—C27A—Cl5	−65.1 (12)
C7—C8—C9—C10	−175.2 (3)	C27B—Cl7A—C27A—Cl6A	56.1 (12)
C8—C9—C10—N2	−161.5 (3)		

### Hydrogen-bond geometry (Å, º)

**Table:**

*D*—H···*A*	*D*—H	H···*A*	*D*···*A*	*D*—H···*A*
N3—H3···Cl4	0.80 (4)	2.60 (4)	3.378 (3)	167 (3)
C27*A*—H27*A*···Cl2^i^	1.00	2.41	3.30 (2)	149

Symmetry code: (i) −*x*+1/2, −*y*+1, *z*+1/2.
